# A new genus and species of berothids (Insecta, Neuroptera) from the Late Cretaceous Myanmar amber

**DOI:** 10.3897/zookeys.864.35271

**Published:** 2019-07-18

**Authors:** Qiang Yang, Chaofan Shi, Dong Ren

**Affiliations:** 1 School of Life Sciences, Guangzhou University, #230 Waihuanxi Road, Guangzhou Higher Education Mega Center, Guangzhou 510006, China Guangzhou University Guangzhou China; 2 School of Earth Sciences and Engineering, Sun Yat-sen University, Guangzhou 510275, China Sun Yat-sen University Guangzhou China; 3 College of Life Sciences, Capital Normal University, Xisanhuanbeilu 105, Haidian District, Beijing 100048, China Capital Normal University Beijing China

**Keywords:** Beaded lacewing, Burmese, fossil, long scape, Mesozoic

## Abstract

A new genus and species of Berothidae is described from the Late Cretaceous (Cenomanian) Myanmar amber. *Ansoberothajiewenae***gen. et sp. nov.** can be easily distinguished from other berothid genera by the long antenna, the scape with ca. 100 flagellomeres, the forewing with four ra-rp, MPand CuA are pectinately branched, and the hind wing with one oblique cua-cup between CuA stem and the distal branch of CuP.

## Introduction

Berothidae is a small family of Neuroptera, comprising approximately 110 extant species assigned to 24 genera, which were divided into six subfamilies (Aspöck and Randolf 2014, Oswald 2019). The family are distributed over all the biogeographic realms except for the Oceania and Antarctica. They are mainly restricted to the tropics and subtropics, with a few occurring in the temperate zone between 50°. Berothidae form a neuropteran clade with Rhachiberothidae and Mantispidae, although the phylogenetic relationships among the three families are still controversial (Tjeder 1959; Willmann 1990; Aspöck and Mansell 1994; Aspöck et al. 2001; Beutel et al. 2010; Zimmermann et al. 2011; Randolf et al. 2013, 2014; Aspöck and Randolf 2014; Haring and Aspöck 2004; Winterton et al. 2010; Engel et al. 2018). In particular, the disagreement on the familial status of Rhachiberothidae resulted in the questionable assignment of the extinct subfamily Paraberothinae (Nel et al. 2005; Makarkin and Kupryjanowicz 2010; Makarkin 2015). Herein, we tentatively follow the cladograms of Neuroptera in Wang et al. (2017), and exclude Rhachiberothidae including Paraberothinae from the family Berothidae.

Berothidae have a fossil history dating back to the Middle Jurassic. Approximately 22 genera with 33 species have been described, mainly distributed in the Eurasia, North and South America (as shown in Table 1). Among them, ten genera with 13 species have been described from the Myanmar amber, representing the most abundant and diverse morphology of the fossil berothids (Engel 2004; Engel and Grimaldi 2008; Yuan et al. 2016; Makarkin 2018; Huang et al. 2019). Herein, a new genus and species of Berothidae is described from the Late Cretaceous Myanmar amber.

**Table 1.** List of named fossil Berothidae.

Species	Age	Locality	Reference
*Sinosmylitespectinatus* Hong, 1983	Middle Jurassic Bathonian to Callovian	Inner Mongolia, China (Jiulongshan Formation)	Hong 1983
*Sinosmylitesfumosus* Makarkin, Yang & Ren, 2011	Middle Jurassic Bathonian to Callovian	Inner Mongolia, China (Jiulongshan Formation)	Makarkin et al. 2011
*Sinosmylitesrasnitsyni* Makarkin, Yang & Ren, 2011	Middle Jurassic Bathonian to Callovian	Inner Mongolia, China (Jiulongshan Formation)	Makarkin et al. 2011
*Berothoneprotea* (Panfilov, 1980)	Upper Jurassic Upper Callovian–Kimmeridgian	Karatau, Kazakhstan (Karabastau Formation)	Khramov 2015
*Berothonegracilis* (Panfilov, 1980)	Upper Jurassic Upper Callovian–Kimmeridgian	Karatau, Kazakhstan (Karabastau Formation)	Khramov 2015
*Krokhathoneparva* Khramov, 2015	Upper Jurassic Upper Callovian–Kimmeridgian	Karatau, Kazakhstan (Karabastau Formation)	Khramov 2015
*Krokhathonetristis* Khramov, 2015	Upper Jurassic Upper Callovian–Kimmeridgian	Karatau, Kazakhstan (Karabastau Formation)	Khramov 2015
*Sinosmyliteskaratavicus* Khramov, 2015	Upper Jurassic Upper Callovian–Kimmeridgian	Karatau, Kazakhstan (Karabastau Formation)	Khramov 2015
*Sinosmylitesauliensis* Khramov, 2015	Upper Jurassic Upper Callovian–Kimmeridgian	Karatau, Kazakhstan (Karabastau Formation)	Khramov 2015
*Sinosmyliteshotgoricus* Khramov, 2015	Upper Jurassic	Khoutiyn-Khotgor, Mongolia (Ulan-Ereg Formation)	Khramov 2015
*Epimesoberothaparva* Jepson, Makarkin & Coram	Early Cretaceous Early Berriasian	Durlston Bay, England (Lulworth Formation)	Jepson et al. 2012
*Banoberothaenigmatica* Whalley, 1980	Early Cretaceous Valanginian/Hauterivian	Lebanese amber (Jezzine)	Whalley 1980
*Sibelliberotharihanensis* Azar & Nel, 2013	Early Cretaceous Valanginian/Hauterivian	Lebanese amber (Jezzine)	Azar and Nel 2013
*Oloberothasinica* Ren & Guo, 1996	Early Cretaceous Barremian	Liaoning, China (Yixian Formation)	Ren and Guo 1996
*Ansoberothajiewenae* gen. & sp. n.	Late Cretaceous lowermost Cenomanian	Myanmar amber	This paper
*Dasyberothaeucharis* Engel & Grimaldi, 2008	Late Cretaceous lowermost Cenomanian	Myanmar amber	Engel and Grimaldi 2008
*Ethiroberothaelongata* Engel & Grimaldi, 2008	Late Cretaceous lowermost Cenomanian	Myanmar amber	Engel and Grimaldi 2008
*Haploberothacarsteni* Makarkin, 2018	Late Cretaceous lowermost Cenomanian	Myanmar amber	Makarkin 2018
*Haploberothapersephone* Engel & Grimaldi, 2008	Late Cretaceous lowermost Cenomanian	Myanmar amber	Engel and Grimaldi 2008
*Iceloberothakachinensis* Engel & Grimaldi, 2008	Late Cretaceous lowermost Cenomanian	Myanmar amber	Engel and Grimaldi 2008
*Iceloberothasimulatrix* Engel & Grimaldi, 2008	Late Cretaceous lowermost Cenomanian	Myanmar amber	Engel and Grimaldi 2008
*Jersiberothamyanmarensis* Engel & Grimaldi, 2008	Late Cretaceous lowermost Cenomanian	Myanmar amber	Engel and Grimaldi 2008
*Jersiberothatauberorum* Engel & Grimaldi, 2008	Late Cretaceous lowermost Cenomanian	Myanmar amber	Engel and Grimaldi 2008
*Protoberothaminuta* Huang, Ren & Wang, 2019	Late Cretaceous lowermost Cenomanian	Myanmar amber	Huang et al. 2019
*Systenoberothamagillae* Engel & Grimaldi, 2008	Late Cretaceous lowermost Cenomanian	Myanmar amber	Engel and Grimaldi 2008
*Telistoberothalibitina* Engel & Grimaldi, 2008	Late Cretaceous lowermost Cenomanian	Myanmar amber	Engel and Grimaldi 2008
*Maculaberothanervosa* Yuan, Ren & Wang, 2016	Late Cretaceous lowermost Cenomanian	Myanmar amber	Yuan et al. 2016
*Magniberotharecurrens* Yuan, Ren & Wang, 2016	Late Cretaceous lowermost Cenomanian	Myanmar amber	Yuan et al. 2016
*Jersiberothaluzzii* Grimaldi, 2000	Late Cretaceous Turonian	Raritan (New Jersey) amber	Grimaldi 2000
*Jersiberothasimilis* Grimaldi, 2000	Late Cretaceous Turonian	Raritan (New Jersey) amber	Grimaldi 2000
*Nascimberothapicta* Grimaldi, 2000	Late Cretaceous Turonian	Raritan (New Jersey) amber	Grimaldi 2000
*Microberothamacculloughi* Archibald & Makarkin, 2004	Early Eocene	Hat Creek amber, British Columbia	Archibald and Makarkin 2004
*Elektroberothagroehni* Makarkin & Ohl, 2015	Late Eocene	Baltic amber	Makarkin and Ohl 2015
*Xenoberothaangustialata* Makarkin, 2017	Early Eocene late Ypresian	Colorado, USA (Green River Formation)	Makarkin 2017

## Materials and methods

This study is based on one female specimen from Myanmar amber. The amber pieces were collected in the Hukawng Valley (the state of Kachin in northern Myanmar). A detailed map of the Hukawng Valley is given by Grimaldi et al. (2002: fig. 1). The volcanoclastic matrix of the amber is estimated to be ~98.79 ± 0.62 million years old, i.e., near the Albian/Cenomanian (Early/Late Cretaceous) boundary (Shi et al. 2012). The biological inclusions of Myanmar amber represent a sample of a tropical forest community in equatorial southeastern Asia at ~12°N paleolatitude (Grimaldi et al. 2002; Poinar et al. 2008; Zhang et al. 2018; Chen et al. 2019; Lin et al. 2019). The specimen was deposited by Ms Dan Zuo in the collections of the Key Laboratory of Insect Evolution & Environmental Changes, College of Life Sciences, Capital Normal University, Beijing, China (**CNUB**; Dong Ren, Curator). The specimen was examined using a Zeiss Discovery V20 stereomicroscope and photographed with an AxioCam HRc digital camera attached to the Zeiss Discovery V20 stereomicroscope (both instruments Carl Zeiss Light Microscopy, Göttingen, Germany). Line drawings were prepared with the Adobe Illustrator CS6 and with the aid of Adobe Photoshop CS6.

Venational terminology generally follows Kukalová-Peck and Lawrence (2004) as interpreted by Yang et al. (2012, 2014). Terminology of details of venation (e.g., spaces, veinlets, traces) follows Oswald (1993). Crossveins are designated after the longitudinal veins with which they connect and are numbered in sequence from the wing base.

Abbreviations:

**AA1–AA3** first to third anterior anal vein;

**CuA** anterior cubitus;

**CuP** posterior cubitus;

**MA** / **MP** anterior and posterior branches of media;

**RA** anterior radius;

**RP** posterior radius;

**RP1** proximal-most branch of RP;

**RP2** branch of RP distal to RP1;

**ScA** subcosta anterior;

**ScP** subcosta posterior.

## Systematic paleontology

### Class Insecta Linnaeus, 1758

#### Order Neuroptera Linnaeus, 1758

##### Family Berothidae Handlirsch, 1906

###### Genus Ansoberotha gen. nov.

Taxon classificationAnimaliaNeuropteraBerothidae

d4f588ad-2f45-470a-b85a-07487be59d47

http://zoobank.org/A9486E3A-C995-430F-9D54-F45F1DC9279B

####### Type (and only) species.

*Ansoberothajiewenae* gen. et sp. nov.

####### Etymology.

The generic name is a combination of the Latin *ansa* (meaning haft, handle), and *Berotha*, the type genus of the family, in reference to the long scapus. Gender feminine.

####### Diagnosis.

Antenna long, more than 6.6 mm, longer than body or forewings; scape elongate, ca. 0.64 mm, almost 12 times as long as wide; flagellum with about 100 flagellomeres. Pronotum elongate, about three times as long as wide. Forewing with one basal sc-r and four ra-rp, M forked distal to the separation of RP; MP, CuA pectinately branched. Hind wing with one r-m between RP stem and MA; one oblique cua-cup between CuA stem and distal branch of CuP.

###### Ansoberotha jiewenae gen. et sp. nov.

Taxon classificationAnimaliaNeuropteraBerothidae

395e9c89-c9bc-4e4d-ad3a-5a854a1f7789

http://zoobank.org/08878C7B-AC2C-48D8-BD01-824134515A83

Figures 1 , 2

####### Etymology.

The specific epithet is named after Ms Jiewen Zhao (Hunan, China), the daughter of this amber’s owner (Ms Dan Zuo). Her mother hopes that this honour will promote Jiewen’s interests in natural history.

####### Diagnosis.

As for the genus.

####### Holotype.

CNU-NEU-MA2018072, female, a nearly complete and well-preserved specimen.

####### Locality and horizon.

Hukawng Valley, Kachin State, northern Myanmar; lowermost Cenomanian, Upper Cretaceous.

####### Description.

Holotype CNU-NEU-MA2018072. Total body length 4.0 mm. Head and body with numerous scattered, fine setae; head about as wide as long. Compound eyes large. Antenna filiform, over 6.6 mm, with scattered setae all over; scape elongate, ca. 0.64 mm, almost 12 times as long as wide; pedicel as long as wide, slightly thicker than flagellum; flagellum with approximately 100 flagellomeres, the last few flagellomeres tapering. Pronotum elongate, narrower than head, about three times as long as wide; pro-, meso-, and metanotum with scattered, long, fine setae. Legs relatively long and slender, with numerous short setae intermixed with long setae. Forelegs: coxa elongated; femur long and slender; tibia slightly inflated nearly as long as femur; basitarsus nearly three times as long as the second tarsomere, the last four tarsomeres of the same length, each tarsomeres with two ended spur. Mid- and hind legs coxa coniform, thicker than forelegs. Each leg with two pretarsal claws, one big arolium. Abdomen nine segments, with scattered short setae; gonapophysis lateralis elongate.

Forewing length 5.5 mm, width 1.5 mm (left forewing/LFW); length 4.9 mm, width 1.8 mm (right forewing/RFW); elongated ovoid, apex rounded, with dense relatively short setae on veins and longer setae on margins; trichosors prominent along entire wing margin. Humeral vein crossvein-like; presumable ScA not detected; costal space relatively broad; most subcostal veinlets simple, not forked, only three (LFW) or four (RFW) distal apex subcostal veinlets forked once, pterostigma not present. ScP and RA fused distally, entering margin before wing apex; ScP+RA with five forked veinlets. Subcostal space slightly narrower than costal space, basally narrowed; only one sc-r present in right forewing, left forewing not detected due to preservation; four ra-rp crossveins located proximal to the fusion of ScP and RA. RP separated from R distal to sc-r, with six (LFW) or five (RFW) branches; RP4 (LFW) dichotomously forked, RP3 (RFW) pectinately forked, with three branches; only one crossveins detected between RP1, RP2 in LFW. M divided into MA and MP distal to the origin of RP and proximal to the separation of RP1 from RP stem, one ma-mp crossvein present; MA distally pectinately forked, with three branches; MP pectinately forked, with seven (LFW) or six (RFW) branches; two crossveins between stem RP, MA and RP1, MA. Cu divided into CuA and CuP near wing base, with one m-cu detected in LFW, two in RFW; CuA pectinately forked, with five (LFW) or six (RFW) distal forked branches; CuP pectinately forked, with three or four simple branches, one crossvein between CuA, CuP in RFW detected. AA1 with a distal fork; AA2, AA3 not detected; no crossveins detected between AA region. Membrane without colour pattern.

Hind wing elongate, length 5.1 mm, width 1.5 mm (left hind wing/LHW); length 5.2 mm, width 1.5 mm (right hind wing/RHW). Trichosors prominent along entire wing margin. Costal space narrow, dilated distal to the fusion of ScP and RA; subcostal veinlets simple, widely spaced, pterostigma not present. Subcostal space no crossveins detected. ScP and RA fused distally, entering margin before wing apex; ScP+RA with seven (LHW) or five (RHW) veinlets, most with distal fork. RA space wider than subcostal space, with two (LHW) or three (RHW) ra-rp located proximal to the fusion of ScP and RA. RP originated slightly distal to wing base, with five pectinate branches, most forked distally; RP4 of LHW, RP3 of RHW dichotomously forked distally; no crossveins between RP branches; one r-m between RP stem and MA. M forked distal to origin of RP and proximal to the origin of RP1; MA dichotomously branched distally; MP pectinately forked, with six (LHW) or five (RHW) branches, most with distal fork; one ma-mp between MA and MP. Cu divided into CuA and CuP near wing base; with two m-cu detected, one near wing base, another located between RP and CuA branches; CuA long, parallel with the posterior margin, pectinately branched with eight (LHW) or 10 (RHW) simple branches; CuP with three distal simple pectinate branches; one oblique cua-cup between CuA stem and diatal branch of CuP. AA1 with a distal fork; AA2 simple; AA3 not detected; no crossveins detected between AA region. Membrane without colour pattern.

**Figure 1.**
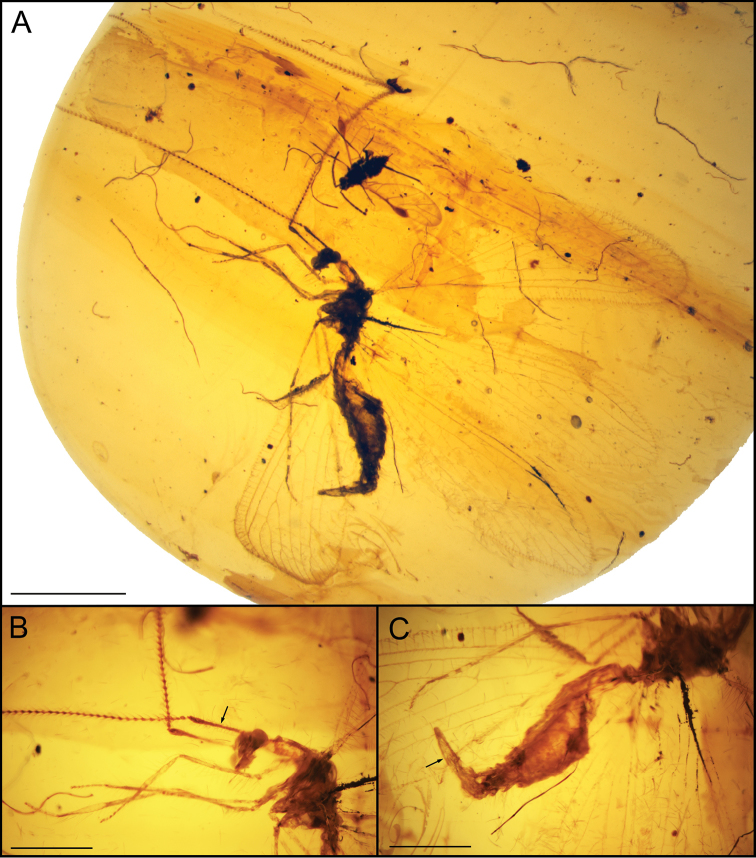
*Ansoberothajiewenae* gen. et sp. nov., holotype CNU-NEU-MA2018072 **A** photograph of holotype **B** detailed photograph of antenna, arrow shows the long scape **C** detailed photograph of abdomen, arrow shows the gonapophysis lateralis. Scale bars 2 mm (**A**) and 1 mm (**B, C**).

**Figure 2.**
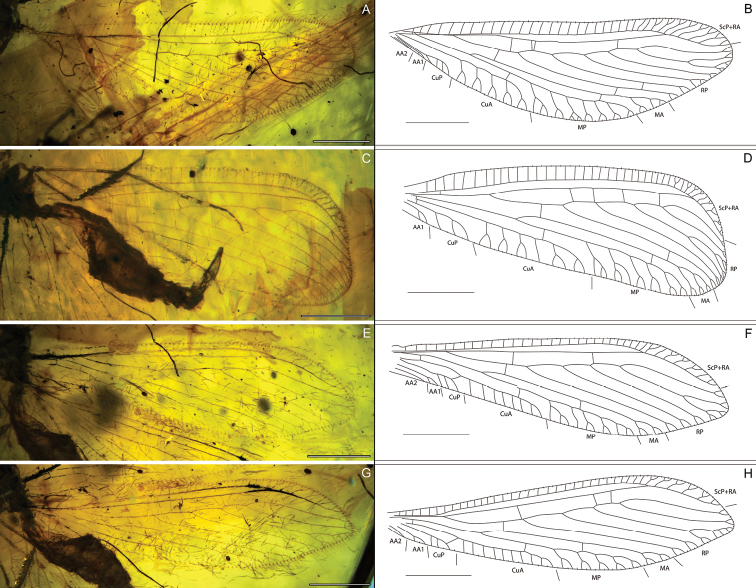
*Ansoberothajiewenae* gen. et sp. nov., holotype CNU-NEU-MA2018072 **A, B** photograph of left forewing and line drawing **C, D** photograph of right forewing and line drawing **E, F** photograph of left hind wing and line drawing **G, H** photograph of right hind wing and line drawing. Scale bars 1 mm.

####### Remarks.

*Ansoberotha* gen. nov. is distinctly different from the other Burmese amber berothid genera by having following characters: (1) *Ansoberotha* gen. nov. antenna is very long, over 6.6 mm, longer than body or forewings; the scape is elongate, ca. 0.64 mm, almost 12 times as long as wide; the flagellum with approximately 100 flagellomeres; other genera without such long antenna, scape, or so many flagellomeres; (2) the forewing of *Ansoberotha* gen. nov. with four ra-rp; *Ethiroberotha* and *Protoberotha* without ra-rp; *Haploberotha* and *Maculaberotha* with only one ra-rp; *Jersiberotha*, *Iceloberotha*, *Telistoberotha*, and *Dasyberotha* with two ra-rp; (3) the forewing MP and CuA are pectinately branched, with no less than five branches; (4) the hind wing of *Ansoberotha* gen. nov. with one oblique cua-cup between CuA stem and the distal branch of CuP; other genera do not have this crossvein.

## Supplementary Material

XML Treatment for Ansoberotha

XML Treatment for Ansoberotha jiewenae
